# Birth Weight, Birth Length, and Gestational Age as Indicators of Favorable Fetal Growth Conditions in a US Sample

**DOI:** 10.1371/journal.pone.0153800

**Published:** 2016-04-20

**Authors:** Marie Camerota, Kenneth A. Bollen

**Affiliations:** 1 Department of Psychology and Neuroscience, The University of North Carolina at Chapel Hill, Chapel Hill, North Carolina, United States of America; 2 Department of Psychology and Neuroscience and Department of Sociology, The University of North Carolina at Chapel Hill, Chapel Hill, North Carolina, United States of America; University of São Paulo, BRAZIL

## Abstract

The “fetal origins” hypothesis suggests that fetal conditions not only affect birth characteristics such as birth weight and gestational age, but also have lifelong health implications. Despite widespread interest in this hypothesis, few methodological advances have been proposed to improve the measurement and modeling of fetal conditions. A *Statistics in Medicine* paper by Bollen, Noble, and Adair examined favorable fetal growth conditions (FFGC) as a latent variable. Their study of Filipino children from Cebu provided evidence consistent with treating FFGC as a latent variable that largely mediates the effects of mother’s characteristics on birth weight, birth length, and gestational age. This innovative method may have widespread utility, but only if the model applies equally well across diverse settings. Our study assesses whether the FFGC model of Cebu replicates and generalizes to a very different population of children from North Carolina (N = 705) and Pennsylvania (N = 494). Using a series of structural equation models, we find that key features of the Cebu analysis replicate and generalize while we also highlight differences between these studies. Our results support treating fetal conditions as a latent variable when researchers test the fetal origins hypothesis. In addition to contributing to the substantive literature on measuring fetal conditions, we also discuss the meaning and challenges involved in replicating prior research.

## Introduction

Few hypotheses have received more attention than Barker’s fetal origins hypothesis [1], both within the current journal [2, 3] and in the fields of medicine and social science more broadly [4]. Much of the existing work is focused on replicating Barker’s original finding that birth weight is inversely related to adult risk of cardiovascular disease, using different populations, different health outcomes, or both. Almost all studies of which we are aware use birth weight as a proxy variable for fetal growth conditions. Despite sustained interest in Barker’s hypothesis over the past two decades, few methodological advances have been proposed to improve our measurement and modeling of fetal growth conditions. A notable exception is recent work by Bollen, Noble, and Adair [5], which demonstrates a latent variable approach to modeling favorable fetal growth conditions (FFGC). FFGC are not directly observable or measurable, which implies that they are latent. It is this latent variable that is the force behind the fetal origins hypothesis. Given the potentially large impact of this improved measurement and modeling approach, the current study examines whether the FFGC latent variable model replicates and generalizes to a different country and time period.

**Fig 1:**
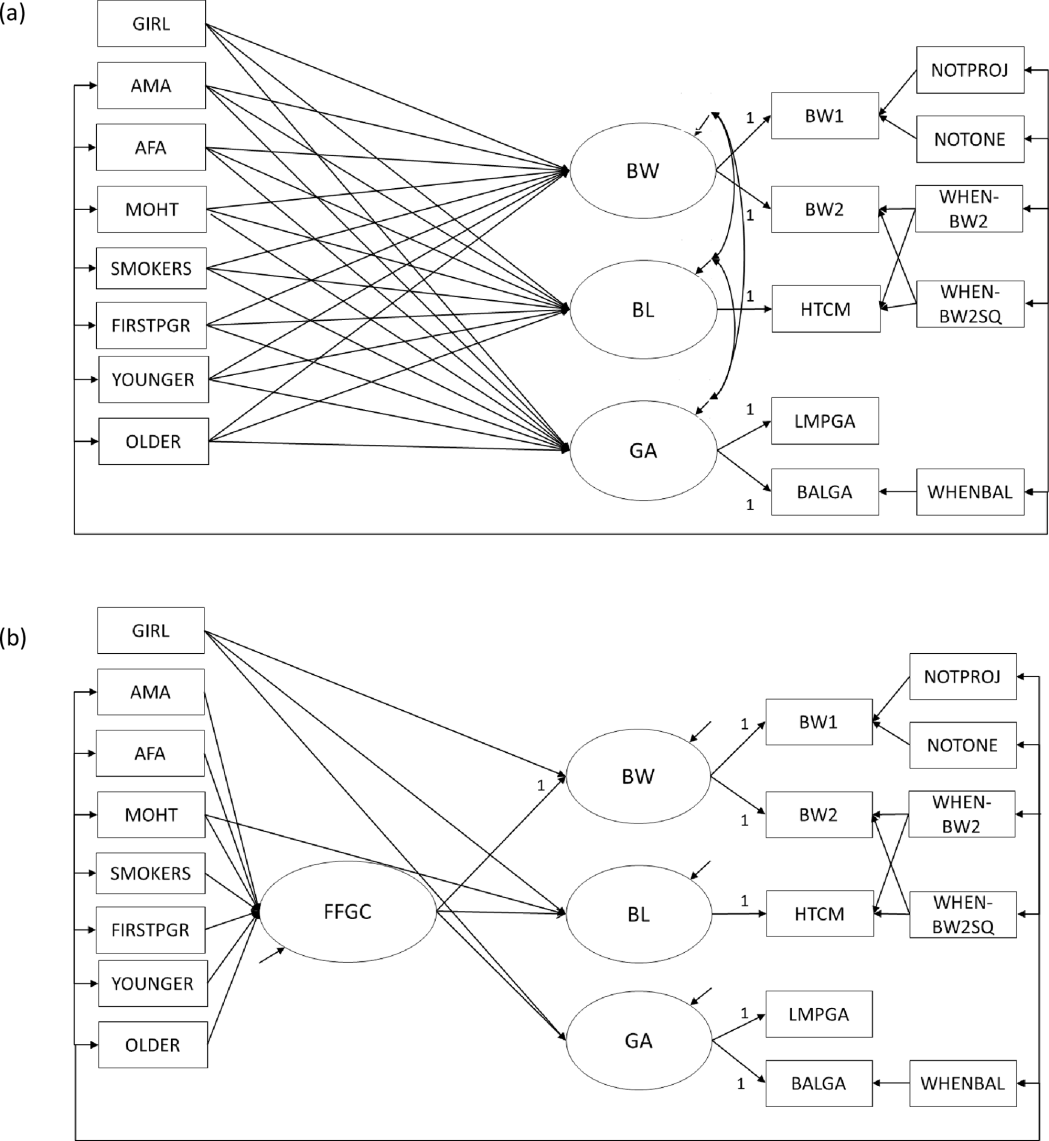
Structural Equation Models from Cebu Analyses. Structural equation model depicting (a) direct-effects only model (Model 1) and (b) favorable fetal growth conditions (FFGC) latent variable model (Model 2) for Cebu. BW = latent newborn weight; BL = latent newborn length, GA = latent gestational age; BW1 = newborn weight measured by birth attendants; BW2 = newborn weight measured by study staff; HTCM = newborn length; LMPGA = gestational age estimated from mother's report of date of her last menstrual period; BALGA = gestational age estimated from Ballard assessment of newborn; NOTPROJ = newborn not weighed on project scale; NOTONE = weight not measured day of birth; WHENBW2 = infant age in days when measured by study staff; WHENBW2SQ = WHEN2BW squared; WHENBAL = age in days when Ballard assessment was done; NOTONE = newborn not weighed on day 1; GIRL = newborn is a girl; AMA = maternal arm muscle area during pregnancy; AFA = maternal arm fat area during pregnancy; MOHT = mother's height; SMOKERS = mother smoked during pregnancy; FIRSTPRG = newborn was firstborn; YOUNGER = mother was <20 years old when pregnant; older = mother was >35 years old when pregnant. (Figures adapted from Bollen et al. [5], p. 13–14).

The fetal origins hypothesis [1], suggests that favorable (or unfavorable) fetal growth conditions have life-long health consequences for outcomes such as adult blood pressure [6, 7] and diabetes risk [8]. Favorable fetal growth conditions (FFGC) is an abstract variable that encompasses all of the environmental, genetic, and epigenetic factors that program prenatal development. It is FFGC that is hypothesized to affect adult health outcomes. However, little time has been devoted to testing whether FFGC exist. Until now, empirical analyses have tended to use birth weight as a proxy for fetal conditions, assuming rather than testing the plausibility of a FFGC latent variable. The use of a single observed measure as a proxy variable is problematic, as this approach assumes that birth weight is a perfectly reliable indicator of fetal conditions, thus ignoring any possible measurement error. In their original analyses, Bollen et al. [5] improve upon this technique by explicitly testing whether FFGC can be modeled as a latent variable, an approach that appropriately accounts for measurement error in each observed indicator. A key result from their analyses is that a model with a FFGC latent variable mediating the effects of maternal characteristics on birth outcomes (Fig 1b and S1 Table) fits better than a model without it (Fig 1a). Full details on the model specification and variables are in the original publication, but an important characteristic of the model is that birth weight, birth length, and gestational age are indicators of latent FFGC and that most maternal characteristics affect these by influencing FFGC. These results are an important first step in providing evidence for the existence of FFGC.

However, there are reasons to be cautious when interpreting these findings on their own. Bollen et al.’s [5] sample was drawn from a metropolitan region of the Philippines (Cebu). In the interest of applying this model to future studies on the fetal origins hypothesis, it is next important to test whether birth weight, birth length, and gestational age function similarly as indicators of FFGC in different populations. The current study tests whether there is evidence of a FFGC latent variable in a sample of mother-infant dyads drawn from two different states, North Carolina and Pennsylvania. While many variables are the same across the Cebu and the US samples, important differences include the industrialization status (developing versus developed), culture (Asian versus Western), the source of maternal and infant data (prospective measurement versus retrospective report; Table 1), and the decade in which the births occurred (1980s versus 2000s). Due to these differences in population, time period, and variables, the results reported here represent a rigorous test of the FFGC model, as we are assessing the degree to which Bollen et al.’s [5] results both replicate and generalize while adding information on the treatment of FFGC as a latent variable in these different contexts.

**Table 1:** Descriptive Statistics for Cebu and NC/PA.

			Cebu			NC			PA		
Infant’s Variables		Source	N	Mean or Proportion	SD	N	Mean or Proportion	SD	N	Mean or Proportion	SD
Birth Weight, g	BW	Maternal report				689	3287	531	486	3306	511
	BW1	Measured at place of delivery	2615	3028	472						
	BW2	Measured by project staff	3031	2994	435						
Birth Length, cm	BL	Maternal report				664	51.20	3.38	484	49.50	2.95
	HTCM	Measured by project staff	3032	49.25	2.11						
Gestational Age, weeks	GA	Maternal report				683	39.25	1.04	481	39.25	1.04
Gestational Age, ln(weeks)							3.67	0.04		3.67	0.04
	BALGA	Ballard Assessment	597	38.86	1.04						
				3.66	0.04						
	LMPGA	LMP Dating	2843	38.86	1.07						
				3.66	0.07						
**Mother’s Variables**											
Maternal Arm Muscle, cm^2^	AMA	Measured during pregnancy	3058	34.10	5.59						
Maternal Arm Fat, cm^2^	AFA	Measured during pregnancy	3058	14.73	5.67						
Maternal Weight, lbs	MOMWT	Maternal report				661	160.4	46.1	476	151.2	41.9
Maternal Weight, kg							72.8	20.9		68.6	19.0
Maternal Height, cm	MOMHT	Maternal report	3059	150.56	5.00	682	164.2	6.93	486	164.1	7.10
Mother was a smoker, proportion	SMOKERS	Maternal report	3059	0.13	0.34	684	0.18	0.38	486	0.32	0.47
First Pregnancy, proportion	FIRSTPRG	Maternal report	3059	0.22	0.42	689	0.38	0.48	486	0.42	0.49
Mother < 20, proportion	YOUNGER	Maternal report	3059	0.13	0.34	689	0.17	0.37	486	0.14	0.34
Mother > 35, proportion	OLDER	Maternal report	3059	0.10	0.30	689	0.06	0.24	486	0.08	0.27
Mother is African American, proportion	AA	Maternal report				690	0.65	0.48	486	0.03	0.18

Gestational age is reported in weeks for ease of interpretation; however, the natural log of gestational age was used in all analyses. Similarly, maternal weight is reported here in pounds and kilograms, although maternal weight in pounds was used in all analyses.

A key question is how we will know whether we have replicated or not. Our approach reflects the idea that there are degrees of replicability. In the context of FFGC, a fundamental aspect of replicating is to test whether a model using FFGC as a latent variable fits as well or better than one without it, as it did for the Cebu data. A second level of replication is whether the signs and significance of the primary coefficients are the same across these different samples. Finally, the highest level of replication tests whether the most important coefficients are of the same magnitude across studies. To the degree that we find evidence of replicability and generalization, we will accumulate evidence that either supports or opposes the plausibility of a FFGC latent variable, which will undoubtedly inform future research and theory on the fetal origins hypothesis.

## Materials and Methods

Data come from the Family Life Project, a longitudinal study conducted in two of the four rural regions of the United States with the highest rates of child poverty [9]. Specifically, three counties in eastern North Carolina (NC) and three counties in central Pennsylvania (PA) were chosen as representing the Black South and Appalachia, respectively. While full recruitment and enrollment details have been documented elsewhere [10], trained research assistants had contact with all women who gave birth in the selected counties between September 2003 and September 2004 (N = 5471). Families were excluded if they did not live in the selected counties, spoke a primary language other than English in the home, or intended to move out of the area in the next three years. These criteria may have resulted in the exclusion of some high-risk families. Of those families eligible to participate, 68% consented, and of these, 58% were invited to participate.

Complex sampling methods utilizing population weight and stratification variables yielded a representative sample of 1,292 families. The current analyses include 1,199 infants in NC (N = 705) and PA (N = 494) where the biological mother was the primary caregiver at 2 months of age. An additional 15 cases in NC and 8 cases in PA were excluded after being identified as multivariate outliers, using a Mahalanobis distance measure. Another 1 case was excluded in NC because the mother’s reported height was more than 5 standard deviations below the mean.

All data on maternal traits and infant birth measures were collected via maternal report at a home visit when infants were 2 months of age. The Institutional Review Board at the University of North Carolina at Chapel Hill approved all data collection activities. Written consent was obtained from primary caregivers at the beginning of the home visit. Data from the Family Life Project may be accessed via the Inter-University Consortium for Political and Social Research [11].

### Mother’s Traits

Trained research assistants conducted structured interviews with mothers at the two month home visit. Mothers reported on their height in feet and inches (MOMHT) as well as their pre-pregnancy weight in pounds (MOMWT). MOMHT was converted to centimeters. Mothers’ height and pre-pregnancy weight were chosen as our indices of maternal nutritional stores, as they were the closest comparable variables to the measures of maternal arm muscle (AMA) and arm fat (AFA) in the Cebu data (S1 Table). Pre-pregnancy weight was chosen, as opposed to pregnancy weight, because the latter measure is potentially confounded with infant birth weight.

Mothers also reported the frequency and number of cigarettes they smoked during each trimester of pregnancy. Smoker status was dichotomized into smokers and non-smokers (SMOKERS), consistent with Bollen et al. [5]. Based on mothers’ self-reported age in years, we created groups of women < 20 years of age (YOUNGER) or > 35 years of age (OLDER). For these dichotomous variables, the referent category was women aged 20–35 years of age. Finally, parity was dichotomized as first pregnancy or not (FIRSTPRG). These four dichotomous variables (SMOKERS, YOUNGER, OLDER, FIRST) were identical in the Cebu and NC/PA samples. Finally, mothers self-reported their primary race as either White or African-American. We dichotomized this variable to represent whether women were African-American (AA) or not, where White women served as the reference group.

### Birth Measures

At the two month home visit, mothers were asked to recall their infant’s birth weight (BW) in pounds and ounces, as well as birth length (BL) in inches. Weight was converted into grams and length was converted into centimeters. To aid model convergence, birth weight was divided by 100 in all analyses. Mothers were also asked to recall their infant’s due date and birth date. Using these two dates, we calculated infant gestational age (GA) in weeks. All three birth outcomes were retained as continuous variables. To reduce skewness and kurtosis, and to remain consistent with the measure of GA in Bollen et al. [5], we used the natural log of GA in our analyses. Infant sex was dichotomized (GIRL), with male infants serving as the reference group.

**Fig 2:**
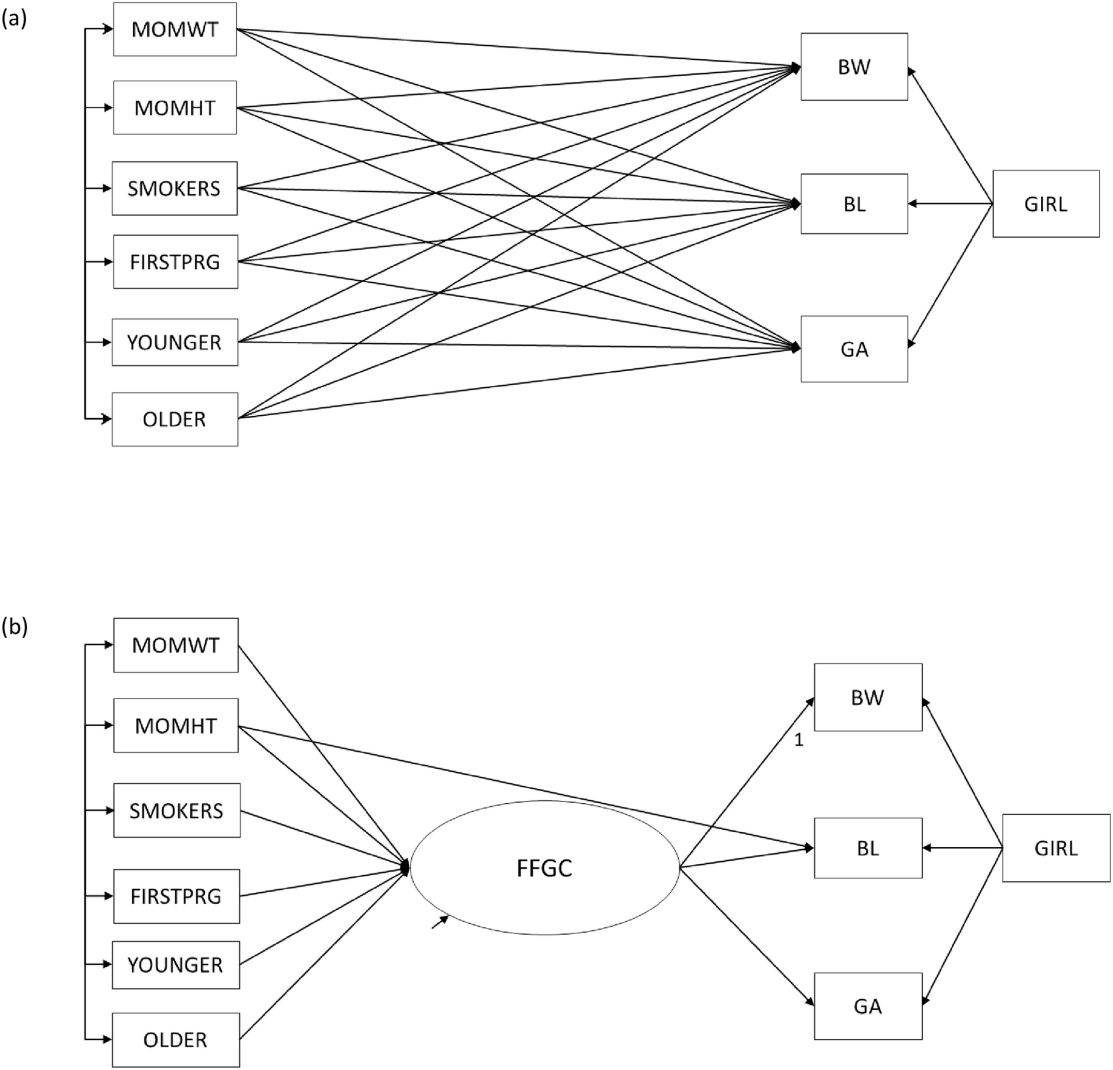
Structural Equation Models from NC/PA Analyses. Structural equation model depicting (a) direct-effects only model (Model 1) and (b) favorable fetal growth conditions (FFGC) latent variable model (Model 2) for NC/PA. BW = birth weight; BL = birth length, GA = gestational age; GIRL = newborn is a girl; MOMWT = mother’s pre-pregnancy weight; MOHT = mother's height; SMOKERS = mother smoked during pregnancy; FIRSTPRG = newborn was firstborn; YOUNGER = mother was <20 years old when pregnant; older = mother was >35 years old when pregnant.

Unlike in the Cebu data, we had only one measure each for BW, BL, and GA in NC/PA. Therefore, whereas in Cebu we could treat BW, BL, and GA as latent variables with multiple indicators (Fig 1), in the following models, they will be manifest, or directly observed, variables (Fig 2). Table 1 provides a comparison of descriptive statistics for all maternal and child characteristics included in analyses in the NC/PA and the Cebu samples.

### Models

A central goal of the current investigation is to test whether BW, BL, and GA are indicators of a common latent variable we call FFGC, as concluded in Bollen et al. [5] or whether they are three distinct outcomes with distinct predictors. If we conclude that a model with a latent FFGC variable fits the data better than a direct-effects only model, then we will move to stricter tests of replication, which includes comparison of signs and significance patterns of coefficients in Cebu and NC/PA, as well as an examination of the magnitude of the factor loadings across samples. Because of the strikingly different contexts of the two samples, we will also explore theoretical and empirical modifications that result in improved model fit, using the NC sample. Finally, we will replicate any modifications using the PA sample, to test whether these additional paths are robust to changes in sample characteristics.

#### Model 1: Direct Effects Model

Our first model allows each predictor variable to have a direct effect on BW, BL, and GA (Fig 2a). Unlike in the Cebu sample, the NC/PA sample has only one indicator of each birth outcome; thus they are treated as manifest, rather than latent variables, and are indicated by rectangles as opposed to ovals. All other observed variables (GIRL, MOMHT, MOMWT, SMOKERS, FIRSTPRG, YOUNGER, OLDER) are exogenous and are allowed to correlate with one another, as indicated by the long bar with the short arrows connecting them. In addition, the set of exogenous observed variables directly influence BW, BL, and GA, as indicated by single-headed arrows. The errors of BW, BL, and GA are also allowed to correlate, to indicate that there is a residual association among them when the impact of maternal variables and GIRL are accounted for.

#### Model 2: FFGC Latent Variable Model

Our second model contains a latent variable (FFGC) that mediates the effect of maternal characteristics on the three birth outcomes (Fig 2b). The existence of the latent variable in this model implies that there is an unobserved variable comprised of the genetic, environmental, and epigenetic conditions that program fetal growth, and which gives rise to our observed measures of BW, BL, and GA. The single-headed arrows from FFGC to BW, BL, and GA indicate that if FFGC increases, we would expect all three birth outcomes to increase; if FFGC decreases, then all birth outcomes would decrease.

To assign a scale to the latent variable, the path from FFGC to BW is set to 1. Like in Bollen et al. [5], GIRL does not have an effect on FFGC; rather, it directly exerts its influence on BW, BL, and GA. Additionally, MOMHT has an effect on FFGC as well as a direct effect on BL, given the likely direct genetic relationship between a woman’s height and the length of her baby at birth.

This model, which is more parsimonious than Model 1, does not allow for correlated errors among BW, BL, and GA; this specification hypothesizes that the association between them is explained by their common dependence on the FFGC latent variable and that there is no residual relationship among the three birth outcomes after we account for FFGC. Like in Model 1, all exogenous variables are allowed to correlate, with the exception of GIRL.

#### Model 3: Modified FFGC Latent Variable Model

Using the NC data, we explored theoretical and empirical modifications to Model 2. Theoretical modifications were made based on the different contexts of births in the US, as opposed to in Cebu. The main theoretically modification explored was the addition of maternal race; while the NC/PA sample included both European American and African American women, the Cebu sample did not. As African American race is associated with higher rates of low birthweight and preterm births in the US [12], we tested whether including African American race (AA) as an additional predictor of birth outcomes improved model fit. AA was allowed to directly influence BW, BL, and GA, instead of having indirect effects via FFGC, because of a lack of theory suggesting why women of AA race would have poorer fetal growth conditions overall.

**Fig 3:**
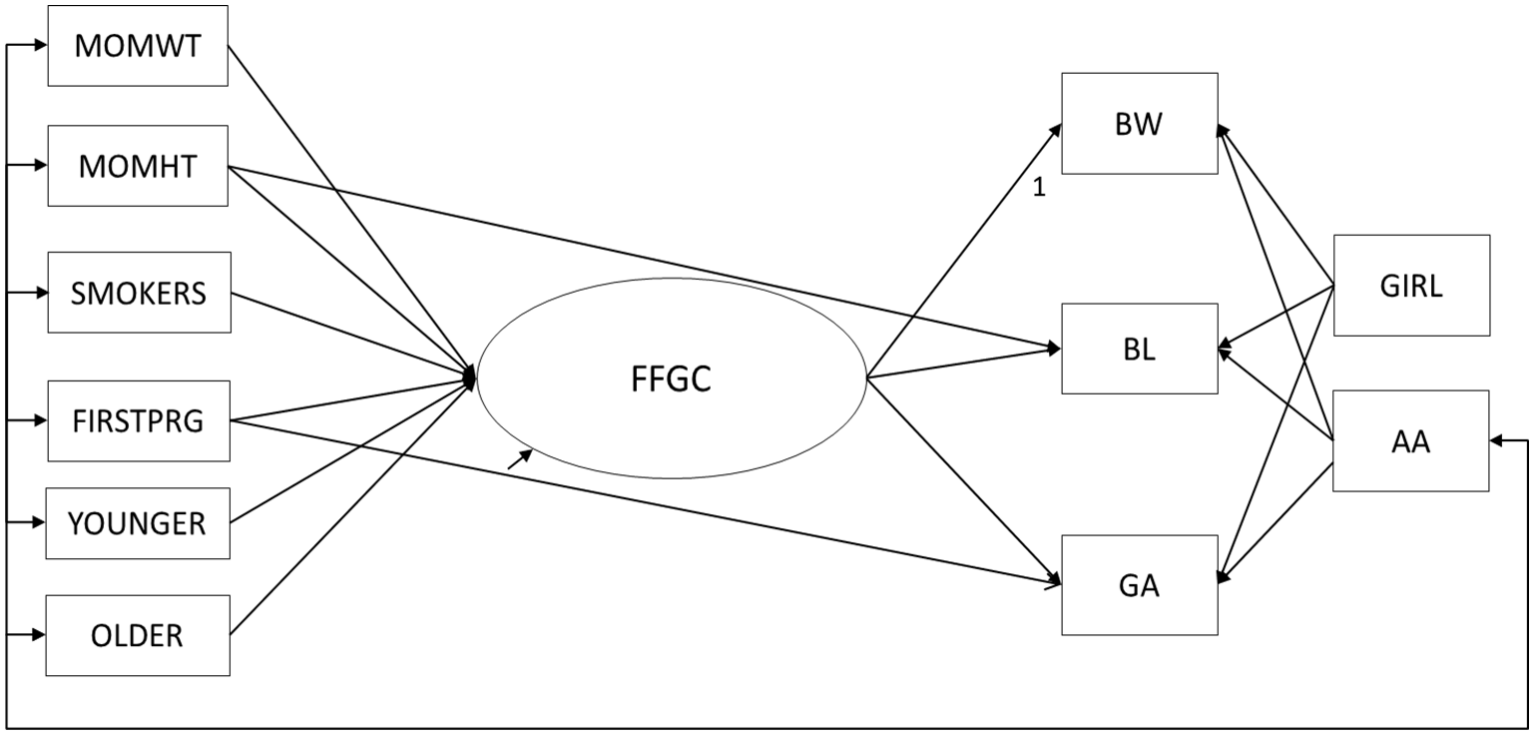
Modified Structural Equation Model from NC/PA Analyses. Structural equation model relating mother’s traits to birth outcomes through the mediating favorable fetal growth conditions (FFGC) latent variable, following theoretical and empirical modifications (Model 3). BW = birth weight; BL = birth length, GA = gestational age; GIRL = newborn is a girl; MOMWT = mother’s pre-pregnancy weight; MOHT = mother's height; SMOKERS = mother smoked during pregnancy; FIRSTPRG = newborn was firstborn; YOUNGER = mother was <20 years old when pregnant; older = mother was >35 years old when pregnant; AA = mother is African-American.

Empirical modifications were also explored, using modification indices (MI) provided by statistical software (Mplus) [13]. While all MI with values above 10 were requested, we only considered modifications that were theoretically justifiable. Although MI are a useful tool for detecting omitted paths, they are also data driven, and must be used with caution [14, 15]. Therefore, any empirical modifications were evaluated carefully to ensure that they were substantively plausible. The only plausible path suggested by this method was the addition of a direct path from FIRST to GA (Fig 3), which is consistent with the finding that primiparous women in the U.S. are more likely to carry their infants past their due date [16].

As an additional test of the robustness of the added paths in the model for NC, we re-ran Model 3 using the PA sample. Because the data from PA are independent from the NC data, this strategy allows us to assess the generalizability of the NC modifications. If the modified model shows similar fit in the PA sample, we next plan to compare the estimates from NC and PA using a multiple group analysis to quantify the extent of agreement between the two samples. Finally, we will interpret the coefficients from the best fitting model, first comparing the patterns of signs and significance of model parameters, and finally testing the statistical equivalence of key model parameters across NC/PA and Cebu.

## Results

All models were estimated in Mplus [13] using full-information, robust maximum likelihood (MLR) as our estimator. MLR was chosen because it is distributionally robust, allowing for possible non-normality in the errors of the model. The scaled chi-square test statistic that results from MLR cannot be used for chi-square difference testing in the normal manner. Therefore, an adjusted calculation was used when comparing nested models in future analyses [17]. Our full-information estimation technique makes use of all cases that have at least partial data, and assumes that any missing data are missing at random (MAR), a less restrictive assumption than missing completely at random. In addition, we have a relatively small proportion of data missing (Table 1).

Descriptive statistics for Cebu, NC, and PA are presented in Table 1. Babies in the NC and PA samples tended to have higher BW than those in Cebu, but mean values of BL and GA were within sampling fluctuation of one another across the samples. Turning towards mother’s characteristics, mothers in the US tended to be taller, and were more likely to smoke, compared to their counterparts in Cebu. Finally, mothers in NC were much more likely to be African American compared to mothers in PA (48% versus 3%). It was not possible to compare mean levels of maternal nutritional stores across the two samples, since different variables were used in Cebu and NC/PA (AMA, AFA, MOMWT).

Our first model comparison addressed whether a model with a mediating FFGC latent variable fit the data better than a model with mother’s characteristics directly influencing birth outcomes. Fig 1a and 1b present Model 1 and Model 2 in Cebu, while Fig 2a and 2b present Model 1 and Model 2 in NC/PA. For this first comparison, we attempted to keep our model as similar to the Cebu analyses as possible. Table 2 contains comparisons of overall model fit statistics for Cebu, NC, and PA. Like in Bollen et al. [5], we report the MLR chi-square test statistic with its corresponding degrees of freedom (df) and p-value, as well as the IFI, [1-RMSEA], and BIC. A non-significant chi-square, a value of IFI and [1-RMSEA] close to 1, and a large, negative BIC value all indicate good model fit.

**Table 2:** Global Fit Measures for Structural Equation Models from NC/PA Analyses.

	Test Statistic	df	*p*-value	IFI	(1-RMSEA)	BIC
*Model 1*						
Cebu	118.569	40	<.001	0.994	0.975	-202.47
NC	0	0	0	0	0	0
PA	0	0	0	0	0	0
*Model 2*						
Cebu	169.006	65	<.001	0.993	0.997	-352.67
NC	57.443	11	<.001	0.842	0.919	-13.82
PA	44.147	11	<.001	0.877	0.920	-23.63
*Model 3*						
NC	24.734	10	<.01	0.942	0.952	-40.08
PA	36.456	10	<.001	0.880	0.925	-25.20
NC/PA	68.675	30	<.001	0.946	0.952	-142.20

The notation NC/PA is used to denote model results from the multiple group analysis.

Turning to the overall fit statistics for Model 1 and Model 2, several points are apparent. As opposed to the Cebu sample, which had multiple indicators of BW and GA, NC/PA had only one indicator each for BW, BL, and GA. As a result, Model 1 is fully saturated in both NC and PA, which means that all available degrees of freedom are used up. All other fit statistics are inapplicable as well, since they are derived from calculations that are based on degrees of freedom. Because a saturated model imposes no restrictions on the data, the model-estimated parameters will perfectly reproduce the covariance matrix of the data. However, this seemingly perfect fit to the data is true for any saturated model and tells us nothing about the overall fit.

In contrast to Model 1, Model 2 is overidentified, with 11 degrees of freedom with which to judge the fit of the model. In both the NC and PA samples, we obtain statistically significant chi-square test statistics, which is typical in moderate to large sample sizes (N = 689 and N = 486 in NC and PA, respectively). Both our IFI and our [1-RMSEA] values are below their ideal fit of 1. However, both models have negative BIC values. According to the Jeffreys-Raftery guidelines [18], a BIC value that is negative and larger in magnitude than 10 suggests strong evidence in support of the model, compared to a saturated model. Using this guideline, Model 2 provides good fit to the data in both the NC and PA samples, which have BICs of -13 and -23, respectively.

Given the mixed evidence provided by the overall fit statistics, we next consider modifications to Model 2. As discussed previously, the first modification we considered was the addition of a direct path from AA to BW, BL, and GA, consistent with the notion that AA women are at higher risk of low birthweight and premature births. Next, an examination of the empirical modifications suggested that we allow a direct path from FIRST to GA. Model 3 was estimated with these two modifications, first in NC, and then in PA, as a check of robustness (Fig 3).

Fit statistics for Model 3 are presented in Table 2. The addition of these paths improved model fit in NC. Although the chi-square test statistic is still significant, both the IFI and [1-RMSEA] are closer to their ideal fit of 1. Additionally, the BIC for Model 3 is even more negative than for Model 2. The paths from AA to BW (β = -3.414), BL (β = -1.123), and GA (β = -.001), as well as the path from FIRST to GA (β = .015) are also all significant at *p* < .05, indicating that the modifications were empirically justified.

In order to verify that the additions made to Model 3 were robust, we replicated our findings using the PA sample. Since the PA sample was not used to estimate the empirical modifications, it served as an independent check of the effect of the added paths. As seen in Table 2, Model 3 also fit the PA data better than Model 2. However, in PA, only the added paths from AA to BW (β = -1.726) and FIRST to GA (β = .006) were significant at *p* < .05. It is worth noting that the proportion of AA women in PA was small (3%) as compared to in NC, where AA women made up almost half the sample (46%). The small proportion of AA women in PA likely contributed to larger standard errors, leading to the different patterns of significance between NC and PA.

Finally, we conducted a multiple group analysis to simultaneously fit Model 3 to the NC and PA samples. By testing a series of increasingly restrictive models, we are able to assess the equality of the parameters across groups, which give us a further test of the robustness of Model 3. If the relationship between our variables is truly the same in both samples, then setting our factor loadings, intercepts, and coefficients to be equal should not result in a significant decrement in model fit. Table 3 shows the chi-square change resulting from imposing increasing equality constraints on the NC and PA models. Setting the factor loadings and intercepts of BL and GA to be equal, as well as the paths from each maternal characteristic to FFGC, does not result in a significant decrement in model fit. However, forcing the direct effects of AA, FIRST, and GIRL on BW, BL, and GA to be equal does significantly worsen model fit. Given the differential patterns of significance for the effects of AA on each birth outcome in NC and PA, it is not surprising that imposing this equality constraint significantly worsens model fit. However, the equality of factor loadings, intercepts, and coefficients on FFGC between the groups provides evidence that Model 3 fits both samples adequately well.

**Table 3:** Chi-Squared Difference Testing of Multiple Groups Models.

	*df*	Χ^2^	ΔΧ^2^	*p*
No Constraints	20	61.006		
Factor Loadings	22	63.274	3.359	.186
Factor Loadings + Intercepts	24	63.319	0.021	.986
Factor Loadings + Intercepts + Beta (FFGC)	30	68.675	5.738	.453
Factor Loadings + Intercepts + All Beta	38	134.318	75.439	<.001

Because MLR was selected as the model estimator, chi-square difference testing could not be done in the usual manner. For more information on chi-square difference testing for MLR, see Satorra and Bentler [17].

Complete fit statistics for the multiple group models are presented in Table 2. Although our chi-square test statistic is significant, all other fit statistics (IFI, [1-RMSEA], BIC) indicate good model fit. Moving to interpreting the coefficients of the model, we first examined the patterns of signs and significance for our parameters (see Table 4). In our model, like in Cebu, indices of maternal nutritional stores (AMA, AFA, and MOHT in Cebu; MOWT and MOHT in NC/PA) positively predicted FFGC, while SMOKERS negatively predicted FFGC. However, our measures of parity (FIRSTPRG) and maternal age (YOUNGER, OLDER) did not significantly predict FFGC, as they did in Cebu. Together, our set of covariates explained 8% of the variance in FFGC in NC and 11% of the variance in PA, compared to 11% of the variance in Cebu.

**Table 4:** MLR Estimates of Direct Effects of Mother's Characteristics on Favorable Fetal Growth Conditions (FFGC).

	Cebu	NC/PA
**Exogenous Variable**	β	β
	[95% CI]	[95% CI]
	FFGC	FFGC
Maternal Arm Muscle (AMA)	.049	
	[.022, .076]	
Maternal Arm Fat, cm^2^ (AFA)/	.088	.129
Maternal Weight, lbs (MOMWT)	[.059, .117]	[.054, .204]
Maternal Height, cm (MOMHT)	1.505	.106
	[1.223, 1.787]	[.063, .149]
Mother was a smoker (SMOKERS)	-.835	-2.318
	[-1.262, -.408]	[-3.036, -1.600]
First Pregnancy (FIRSTPRG)	-1.242	-.033
	[-1.642, -.842]	[-.634, .567]
Mother < 20 (YOUNGER)	-.766	.149
	[-1.232, -.300]	[-.735, 1.033]
Mother > 35 (OLDER)	-.121	-.409
	[-.629, .387]	[-1.653, .835]
*R*^*2*^	.110	.082 (NC)
		.109 (PA)

Coefficients for NC/PA are taken from the multiple group analysis. Although the coefficients are set to be equal across NC and PA, the multiple group analysis results in separate *R*^2^ values for each group. AMA was not measured in NC/PA. AFA was measured in cm^2^ in Cebu, but only MOMWT in pounds was measured in NC/PA.

As our final, strictest check of replication, we tested whether the factor loadings of BL and GA from these analyses (Table 5) were statistically equivalent to those obtained in the Cebu analyses. Because the NC/PA and Cebu samples are independent, it was appropriate to use a z-test to calculate the difference in coefficients between the two models, as well as the significance of the obtained z statistic [19]. For GA, the estimated factor loading of .005 was not significantly different than the Cebu factor loading of .004 (z = .196, p > .05). For BL, the estimated factor loading of .496 was significantly larger than the Cebu factor loading of .348 (z = 4.147, p < .05). Put into context, this finding means that for a 1 unit difference in FFGC we would expect a .50 cm difference in birth length in the NC/PA sample and a .35 cm difference in the Cebu sample. Whether this difference is substantively important remains to be determined, but it is a difference which if replicated in future studies would demand further investigation.

**Table 5:** Factor Loadings for FFGC Indicators.

	Cebu		NC/PA		
	λ	*R*^*2*^	λ	*R*^*2*^	*R*^*2*^
**Indicator**	[95% CI]		[95% CI]	(NC)	(PA)
	FFGC		FFGC		
Birth Weight (BW)	1	1.000*	1	.901	.845
	[N/A]		[N/A]		
Birth Length (BL)	.348	.684	.496	.505	.635
	[.315, .381]		[.435, .557]		
Gestational Age (GA)	.004	.710	.005	.332	.299
	[.0033, .0047]		[.004, .005]		

For NC/PA, the factor loadings, along with their 95% confidence intervals (in brackets), are taken from the multiple group analysis. Although the factor loadings are set to be equal across NC and PA, the multiple group analysis results in separate R^2^ values for each group.

*Model resulted in small negative error variance estimate which was set to zero.

### Sensitivity Analyses

Like in Bollen et al. [5], we tested alternative model specifications to assess whether they had a superior fit. We first tested a model that included GA, rather than FFGC, as a mediator between mother’s characteristics and BW and BL. This model fit the data poorly. We also attempted to model a latent GA variable by setting the reliability of its one indicator (measured GA) to various values (e.g. 0.1, 0.3, 0.5). However, a model with latent GA as a mediator would not converge. Finally, we attempted to estimate a model that included both a FFGC latent variable and a direct path from GA to BW and BL; this model similarly would not converge. Therefore, we concluded that a model with FFGC as a latent variable was the most parsimonious and plausible alternative to the direct effects only model.

Because of the stratified sampling design of the Family Life Project, stratification and weight values were assigned to each case in the NC/PA data. While our models were originally estimated without these variables, we re-estimated Model 3 in both NC and PA accounting for stratification and weight, and found our substantive conclusions unchanged. We also re-ran our final analyses including all cases that were previously excluded as outliers. Although model fit decreased with the inclusion of these unusual cases, all substantive conclusions remained the same.

## Discussion

The goal of the current investigation was to test whether Bollen et al.’s [5] FFGC model, a novel approach to studying the fetal origins hypothesis, replicated and generalized to a new sample of infants born in the United States. In doing so, we demonstrated a graded approach to reproducibility, by describing and then proceeding through a series of increasingly strict tests of replication. Based on this series of tests, we conclude that the results first gleaned from a sample of Filipino infants do generalize to a sample of predominantly low-income American infants. Several theoretically-justifiable modifications were made in our analyses in order to improve model fit, but these modifications were small in number and did not lead to dramatic changes in other model parameters (e.g. factor loadings). Importantly, the final model, including all modifications, fit the data equally well in the two states that we tested (North Carolina and Pennsylvania), which suggests that these findings may be robust to variations in sampling characteristics.

Our substantive conclusion on the generalizability of the FFGC approach is promising for future research on the fetal origins hypothesis, as a latent FFGC variable provides a metric with which to quantify the various environmental, genetic, and epigenetic influences on prenatal development that may program later human health. As opposed to the heretofore popular method of using birth weight as a proxy for prenatal conditions, the FFGC approach is unburdened by measurement error and allows researchers to take advantage of three commonly available birth outcomes. Importantly, the current replication demonstrates the feasibility of modeling FFGC when only one measure each of BW, BL, and GA are available, as well as when these birth outcomes are reported by mothers retrospectively during the early postpartum period. Although there may be concerns about the accuracy of maternal report of these variables, previous work has shown no significant differences between hospital records and maternal report of BW and BL [20]. In addition, treating these variables as indicators of FFGC permits random measurement error to enter the error term for each indicator. The versatility of the FFGC latent modeling approach is promising, as it suggests that modeling FFGC may be appropriate under a wide range of methodological scenarios.

The current study also contributes to the methodological literature by proposing and modeling a series of increasingly rigorous tests of replication. Our first step was to examine whether our substantive conclusions were consistent with those of Bollen et al. [5], which found that a model with a latent FFGC variable fit the data better than a model without it. By comparing fit statistics from Model 1 and Model 2, we confirmed that Model 2 provided a better fit to our data, and thus there was evidence for a FFGC latent variable. Next, we examined the signs and significance of crucial coefficients in our model. Because a central goal was to test whether the relationship between FFGC and its indicators functioned similarly across the two samples, we focused on comparing the factor loadings for BW, BL, and GA. Although the coefficient on BW was set to 1 to scale the latent variable, we found that the freely estimated factor loadings for BL and GA were both positive, significant, and similar in magnitude to the results from Cebu. Our final, strictest test of replication was to test the equality of the factor loadings on BL and GA across the two samples. We found that we could not reject the null hypothesis that the factor loadings for GA were equal in NC/PA and Cebu, while this was not true for those for BL. This high, but not perfect level of agreement between the two studies indicates a relatively strong replication success. In light of our success, we encourage researchers to continue to test the generalizability of the FFGC model. If the FFGC model works similarly well in diverse samples, we will accumulate evidence for the existence of a FFGC latent variable, as well as for the fetal origins hypothesis.

As a rule, science is concerned with conducting robust and reliable research. While recent years have seen a growing recognition of the importance of replication studies as well as a more receptive environment to encouraging their publication [21, 22, 23], the quantity and quality of published replication attempts remain low [24]. Contributing to this quandary is a lack of accepted guidelines on what constitutes a successful replication. We are hopeful that future research will adopt a graded approach to replication, as modeled in the current analyses. This attention to robustness is especially important for research on the fetal origins hypothesis, given its possible lifelong implications for human health and development.

In sum, the current study confirms the existence of a latent variable representing favorable fetal growth conditions which underlies the relationship between maternal characteristics and child birth outcomes. However, the current study does not address whether this latent variable predicts adult health outcomes, as would be predicted by the fetal origins hypothesis. Future research should aim not only to confirm the existence of the FFGC latent variable among diverse populations, but also to test the relationship between FFGC and adult risk of metabolic or cardiovascular disease.

## Supporting Information

S1 Table Variables included in Cebu Analysis. (DOCX)
