# Supplementary material for: Birth Weight, Birth Length, and Gestational Age as Indicators of Favorable Fetal Growth Conditions in a US Sample
Source: PLoS One. 2016 Apr 20;11(4):e0153800. doi: 10.1371/journal.pone.0153800 (PMC4838247; doi:10.1371/journal.pone.0153800)
Supplement: S1 Table — (DOCX) [file pone.0153800.s001.docx]

| **Infant’s Variables** | **Description** |
| --- | --- |
| BW1 | Newborn weight at place of delivery, g |
| BW2 | Newborn weight measured by project staff, g |
| HTCM | Newborn length, cm |
| NOTONE | Weight not measured day of birth, proportion |
| NOTPROJ | Infant not weighed on project scale |
| WHENBW2 | Infant age at weighing by project staff, days |
| BALGA | Gestational age from Ballard assessment, weeks |
| LMPGA | Gestational age from last menstrual period date, weeks |
| WHENBAL | Infant age at Ballard assessment, days |
| GIRL | Infant sex=female, proportion |
| **Mother’s Variables** | **Description** |
| MOHT | Height, cm |
| AMA | Arm muscle area during pregnancy at baseline, cm^2^ |
| AFA | Arm fat area during pregnancy at baseline, cm^2^ |
| SMOKERS | Smoked during pregnancy, proportion |
| FIRSTPRG | Primiparous, proportion |
| YOUNGER | Age < 20 years |
| OLDER | Age > 35 years |
